# Ketamine can be produced by *Pochonia chlamydosporia*: an old molecule and a new anthelmintic?

**DOI:** 10.1186/s13071-020-04402-w

**Published:** 2020-10-20

**Authors:** Sebastiao Rodrigo Ferreira, Alan Rodrigues T. Machado, Luís Fernando Furtado, Jose Hugo de S. Gomes, Raquel M. de Almeida, Thiago de Oliveira Mendes, Valentina N. Maciel, Fernando Sergio Barbosa, Lorendane M. Carvalho, Lilian Lacerda Bueno, Daniella Castanheira Bartholomeu, Jackson Victor de Araújo, Elida M. L. Rabelo, Rodrigo Maia de Pádua, Lucia Pinheiro Santos Pimenta, Ricardo Toshio Fujiwara

**Affiliations:** 1 Departamento de Parasitologia, Instituto de Ciências Biológicas, Universidade Federal de Minas Gerais, Av. Antônio Carlos 6627, Belo Horizonte, MG 31270-901 Brazil; 2 Centro de Formação em Ciências da Saúde, Universidade Federal do Sul da Bahia, Praça Joana Angélica, 250, Teixeira de Freitas, BA 45988-058 Brazil; 3 Departamento de Química, Instituto de Ciências Exatas, Universidade Federal de Minas Gerais, Av. Antônio Carlos 6627, Belo Horizonte, MG 31270-901 Brazil; 4 Departamento de Ciências Exatas, Universidade do Estado de Minas Gerais, Unidade João Monlevade, João Monlevade, MG 35930-314 Brazil; 5 Universidade do Estado de Minas Gerais, Unidade Passos, Avenida Juca Stockler, Nossa Sra. das Gracas, 1130, Passos, MG 37900-106 Brazil; 6 Departamento de Produtos Naturais, Faculdade de Farmácia, Universidade Federal de Minas Gerais, Av. Antônio Carlos 6627, Belo Horizonte, MG 31270-901 Brazil; 7 Departamento de Bioquímica e Biologia Molecular, Universidade Federal de Viçosa, Av. Peter Henry Rolfs, s/n, Viçosa, MG 36.570-000 Brazil; 8 Departamento de Medicina Veterinária, Universidade Federal de Viçosa, Av. Peter Henry Rolfs, s/n, Viçosa, MG 36.570-000 Brazil

**Keywords:** *Pochonia chlamydosporia*, New drugs, Nematicidal molecule, Ketamine

## Abstract

**Background:**

Infection by nematodes is a problem for human health, livestock, and agriculture, as it causes deficits in host health, increases production costs, and incurs a reduced food supply. The control of these parasites is usually done using anthelmintics, which, in most cases, have not been fully effective. Therefore, the search for new molecules with anthelmintic potential is necessary.

**Methods:**

In the present study, we isolated and characterized molecules from the nematophagous fungus *Pochonia chlamydosporia* and tested these compounds on three nematodes: *Caenorhabditis elegans*; *Ancylostoma ceylanicum*; and *Ascaris suum*.

**Results:**

The ethyl acetate extract showed nematicidal activity on the nematode model *C. elegans*. We identified the major substance present in two sub-fractions of this extract as ketamine. Then, we tested this compound on *C. elegans* and the parasites *A. ceylanicum* and *A. suum* using hamsters and mice as hosts, respectively. We did not find a difference between the animal groups when considering the number of worms recovered from the intestines of animals treated with ketamine (6 mg) and albendazole (*P* > 0.05). The parasite burden of larvae recovered from the lungs of mice treated with ketamine was similar to those treated with ivermectin.

**Conclusions:**

The results presented here demonstrate the nematicidal activity of ketamine *in vitro* and *in vivo*, thus confirming the nematicidal potential of the molecule present in the fungus *P. chlamydosporia* may consist of a new method of controlling parasites.
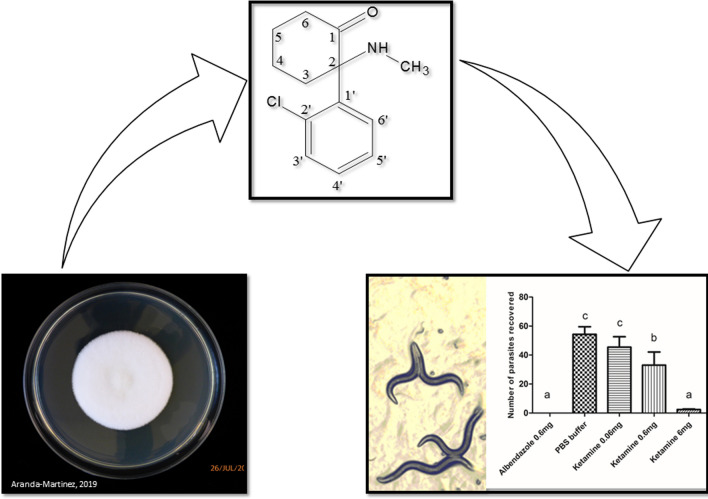

## Background

Nematode infection is a problem for human health, as it contributes negatively to growth and cognitive development. In animal production and agriculture, damages go beyond organic deficits because they incur increased production costs and consequently a reduced food supply [1–3]. The control of these parasites is usually carried out using anthelmintics that, in most cases, have not been totally effective. In addition, resistance to the main group of drugs used in the treatment and control is already a consolidated problem [4]. Thus, the search for new drugs and control alternatives is necessary.

Several studies in the literature have described the direct or indirect nematicidal action of some organisms, such as fungi, featuring one of the alternatives of biological control. The administration of nematophagous fungi is considered a promising alternative in the prophylaxis of parasitic gastrointestinal helminthiasis [5]. Willcox & Tribe [6] reported the association between the fungus *Pochonia chlamydosporia* and the nematode *Heterodera schachtii*, as the primary and secondary metabolites of nematophagous fungi have been known to be involved in this interaction [5, 7, 8]. In a review by Li et al. [9], 179 substances from fungi, including nematophagous, were reported as potential nematicidal drugs; therefore, these fungi can be considered an important source of new drugs.

In the history of drug production and discovery, often a molecule that is characterized with a well-defined function can, with new studies, be employed for a new purpose. This practice is called drug repositioning [10]. In helminthology, some drugs that were not originally used against helminths have shown high efficacy against some worms, such as mefloquine for *Echinococcus multilocularis* [11]. Sertraline, paroxetine, and chlorpromazine have long histories of clinical use as antidepressant or antipsychotic medicines. However, according to Weeks et al. [12], they may represent new classes of anthelmintic drugs. In this perspective, other drugs that act *via* ion channels or neurotransmitter receptors may have promising functions for helminth control. Here, we extracted, characterized, and evaluated *P. chlamydosporia* metabolites against nematodes. In these studies, it is important to have good models to test drug effectiveness. In this context, *Caenorhabditis elegans* is the main model for a drug-screening study, since it presents characteristics that facilitate the assays, such as the feasibility of laboratory maintenance [13] and the taxonomic and functional proximity to important parasitic nematodes [14]. However, validation of the experiments in an animal model is necessary. It is important to consider that we also carried out tests on two parasitic nematodes, *Ancylostoma ceylanicum* and *Ascaris suum*, using the infection model, hamsters and mice, respectively. Therefore, in the present work, we have extracted and characterized a new metabolite of *P. chlamydosporia*: the molecule ketamine. We evaluated this molecule on nematodes, *C. elegans*, *A. suum* and *A. ceylanicum*, and here, we present unprecedented to our knowledge, results of its nematicide activity.

## Methods

### Fungus cultivation

The *P. chlamydosporia* used in this study was kept in the fungal collection of the Veterinary Parasitology Laboratory of the Federal University of Viçosa, Viçosa, Brazil. Culture discs with an approximate diameter of 4 mm were extracted from the tube containing the fungal isolate and then transferred to a 250 ml Erlenmeyer flask containing the potato dextrose liquid medium (Difco, Detroit, USA). These were incubated in a horizontal shaker (Tecnal, Piracicaba, Brazil) at 28 °C under agitation at 120× *rpm*, and, after 20 days, this material was filtered to separate the extract from the mycelial mass. The separated mass was used in the extraction process by maceration.

To obtain the methanolic extract, 400 g of the mycelium (a product of 30 l of fungal culture) had been submitted to cold triple maceration for 7 days in methanol. The methanolic extract (MeOH) was concentrated under reduced pressure and lyophilized, obtaining 4.1 g of methanolic extract. 3 g of this extract were solubilized in MeOH, and then this solution was partitioned with dichloromethane, followed by ethyl acetate, affording the fractions CH_2_Cl_2_ (0.61 g) and EtOAc (0.50 g). 0.4 g of the ethyl acetate fraction (EtOAc) was further fractionated by column chromatography in sub-fractions using silica gel as the stationary phase and then eluted with hexane: ethyl acetate gradients of increasing polarity. These sub-fractions were concentrated under reduced pressure in a rotary evaporator and lyophilized (Table 1).

**Table 1:** Fractions from EtOAc column fractionation

Mobile phase	Mass (g)^a^	Coding name
Hex:EtOAC (9:1)	0.01	F1
Hex:EtOAC (8:2)	0.03	F2
Hex:EtOAC (7:3)	–	F3
Hex:EtOAC (6:4)	0.01	F4
Hex:EtOAC (5:5)	0.04	F5
Hex:EtOAC (4:6)	–	F6
Hex:EtOAC (3:7)	0.06	F7
Hex:EtOAC (2:8)	0.04	F8
Hex:EtOAC (1:9)	0.04	F9

^a^The sub-fractions F3 and F6 of EtOAc were not obtained in sufficient biomass

### Nuclear magnetic resonance (NMR) spectroscopy

The NMR experiments were obtained on a Bruker AVANCE DRX400 spectrometer, at a High-Resolution Magnetic Resonance Laboratory (LAREMAR), of the Chemistry Department-ICEx-UFMG. The fractions were analysed by ^1^H NMR. EtOAc and CH_2_Cl_2_ were solubilized in CDCl_3_ (deuterated chloroform), and MeOH were solubilized in a solution of methanol-*d*_4_/buffered KH_2_PO_4_ in D_2_O, pH 6. The spectra were acquired at 300 K with a spectral window of 16 ppm, a total of 32 k points, 16 intermediate points, acquisition times (AQ), and a recovery (d1) of 2.6 s and 2.0 s, respectively. For the processing, a line widening of 0.3 Hz was used prior to the Fourier transform. The phases and baselines were automatically corrected using the TopSpin 1.3 programme, and, finally, the spectra were calibrated by the TMS signal (Trimethylsilane) at 0.00 ppm, with the exception of the sample spectrum MeOH, which was calibrated by the signal of (3-(trimethylsilyl)propionic-2,2,3,3-*d*_*4*_ acid sodium salt) TSP-*d*_4_ at 0.00 ppm. 2D NMR ^1^H-^1^H COSY (correlated spectroscopy), ^1^H-^13^C HSQC (Heteronuclear Single Quantum Correlation), and ^1^H-^13^C HMBC (Heteronuclear Multiple Bond Correlation) experiments were performed for the F5 sample.

### GC-MS analysis

The ethyl acetate fraction and its 7 sub-fractions obtained from the purification by column chromatography (F1-F9) were analysed by gas chromatography coupled with mass spectrometry (GC/MS). The analyses were performed using a Shimadzu GCMSQP2010 Plus, equipped with an AOC-10 automatic injection system (Shimadzu, Tokyo, Japan). In the experiments, a 30-m-long capillary column Rxi-1 (100% polydimethylsiloxane), with an internal diameter of 0.25 mm and a film thickness of 0.25 μm, was used. The injector temperature was 250 °C, and the temperature programme ranged from 150 °C to 300 °C at 3 °C/min. The injected volume was 1 μl in split-mode at a ratio of 10:1. MS analysis was carried out in a quadrupole MS system (QP-2010plus) operating at 70 eV under the same conditions described above. Compounds were identified through comparison with mass spectral data in NIST 08 libraries.

### UPLC-ESI-MS/MS

4.0 mg of the ethyl acetate fraction and the standard molecule (ketamine) were weighed directly in a microtube and 1 ml of methanol/water (1:1). The HPLC grade was added until reaching complete solubilization. After that, the ethyl acetate sample was diluted 4× and ketamine was diluted 20×, and then they were automatically injected onto the system.

The chemical composition of ethyl acetate fractions was investigated by ultra-performance liquid chromatography coupled with mass spectrometry in a Waters ACQUITY UPLC system (Waters, Milford, MA, USA) composed of a binary pump, auto sampler, in-line degasser, and photodiode array detector (190–500 nm) (Waters). The analyses were performed on an Acquity UPLC BEH C18 column (2.1 × 50 mm i.d., 1.7 µm; Waters) at 40 °C, eluting with a linear gradient of water (A) and acetonitrile (B), both containing 0.1% v/v formic acid, at a flow rate of 0.3 ml/min (e.g. 5 to 95% of B in 10 min) before returning to the initial conditions in 1 min. A re-equilibration time of 2 min was maintained between runs. As previously described, our research group employed the spectroscopic conditions [15].

A mass spectrometer Xeco^TM^ Triple Quadrupole MS (Waters) equipped with an electrospray ionization (ESI) source, operating in a negative and positive ionization mode, was used in the analysis. The cone gas flow was set to 90 L/h, and desolvation gas flow was set to 900 L/h at 350 °C. The capillary voltage was set to 3.54 kV, cone gas voltage was set to 27 V, and source temperature was set to 120 °C. The data were accomplished in the scan mode, with mass range adjusted to *m/z* 100–700 Da. Ketamine (Holliday-Scott^®^, Buenos Aires, Argentine) was employed as the reference compound. The UPLC-ESI-MS/MS analysis was performed twice with the fungus grown at different times for confirmation of ketamine’s presence.

### Culturing *Caenorhabditis elegans* larvae and motility test

The strain of *C. elegans* used in the experiment was kindly provided by Professor Carlos Winter of Universidade de São Paulo (USP). L3 larvae of *C. elegans* were grown on 8P NGM plates according to the methodology previously described [16]. After 7 days of culturing in a bio-oxygen demand (BOD) incubator at 20 °C, the plates were washed with M9 medium [16] and filtered through three sieves with 40-, 30- and 20-μm pores. L3 larvae retained in the 20 μm strainer were collected by backwashing. The obtained larvae were washed by centrifugation at 700×*g* for 4 min, followed by two washes with M9 medium. For the nematicidal assay against *C. elegans*, the L3 was resuspended in M9, and approximately 1000 larvae in 100 μl of suspension were added to each well in a 96-well microplate. The ethyl acetate extract was added in concentrations of 1000, 100, 10, 1, 0.1 and 0.01 μg/ml. For methanol and dichloromethane, concentrations of 5000 and 3000 μg/ml were added, and ketamine was used up until the concentration of 20,000 μg/ml. In the negative control, the M9 medium was used with 0.05% dimethyl sulfoxide, and, for the positive control, ivermectin was applied in the same concentrations used for the tested extracts (1000–0.01 μg/ml). Plates containing compounds and larvae were stored in a BOD incubator at 20 °C. After 48 h, 10 μl of a solution containing approximately 100 larvae were removed from each well for analysis, and quantification of the total number of paralyzed larvae was carried out using an optical microscope at 100× magnification. Larvae were considered paralyzed when presenting a straight body and absence of any mobility.

### Activity of ketamine on *Ancylostoma ceylanicum*

For this experiment, 50 female hamsters (*Mesocricetus auratus*), 4 to 6 weeks-old, were divided into five experimental groups containing 10 animals each. All animals were maintained in a controlled environment and infected with 75 third-stage larvae (L3) of *A. ceylanicum* at day 0 of the experiment. After 20 days of infection, when all animals were excreting eggs in the faeces, they were treated for 5 days, orally, according to the following experimental design: Group 1 PBS (phosphate buffer saline); Group 2 albendazole (Nova Química^®^,Campinas, Brazil) 0.60 mg; Group 3 ketamine 0.06 mg; Group 4 ketamine 0.60 mg; and Group 5 ketamine 6 mg (Holliday-Scott^®^). The eggs per gram of faeces (EPG) was performed to monitor the quantification of eggs throughout the experiment. This quantification was performed every 2 days using the McMaster chamber [17]. After the treatment period, the animals were euthanized using an overdose of anaesthetic (45 mg/kg of xylazine associated with 240 mg/kg of ketamine, intraperitoneally). Then, the small intestine was removed and placed in a Petri dish containing PBS, and it was opened longitudinally for the recovery of adult worms.

### Activity of ketamine on infection of *Ascaris suum* larvae

In this experiment, 60 mice of the C57Bl6 lineage were used, being divided into two groups of 30 animals: a group of 30 animals with treatment administered subcutaneously and the other group by oral route (gavage). The animals had been infected with 2000 eggs of *A. suum* larvae and divided into groups, with 6 animals each, that received the following treatments: Group 1 PBS (phosphate buffered saline); Group 2 ivermectin 0.05 mg (Ouro Fino^®^ Cravinhos, Brazil); Group 3 ketamine 0.05 mg; group 4 ketamine 0.25 mg; and Group 5 ketamine 0.50 mg (Holliday-Scott^®^). The treatment was carried out for 7 days, except for the subcutaneous ivermectin group that received a single dose.

On the eighth day after infection, the animals were euthanized using an anaesthetic overdose (45 mg/kg of xylazine associated with 240 mg/kg of ketamine, intraperitoneally). To recover and quantify the number of larvae present in the lungs, the lung tissue collected was triturated in the Petri dish and placed in the Baermann apparatus for 4 h at 37 °C with PBS. After incubation, the sediment was collected and centrifuged at 600× *g* for 10 min at 20 °C, and the larvae were fixed in 10% formalin and counted in the optical microscope (Leica Microsystems^®^, Wetzlar, Germany) with a 100× magnification.

### Statistical analyses

The data of the recovered nematodes were submitted to the normality test (Kolmogorov-Smirnov), followed by the analysis of variance (ANOVA) and Tukey’s *post-hoc* test (P ≤ 0.05). The EPG data were analyzed using two-way ANOVA and Bonferroni correction (P ≤ 0.01). Nonlinear regression analysis was used to calculate the ED_50_ value utilized of a four-parameter sigmoid curve. All the statistical analyses were carried out in GraphPad Prism 7.0.

## Results

### Anthelmintic activity

To evaluate the nematicidal activity of *P. chlamydosporia*, we tested the extracts obtained from the fungus on *C. elegans* larvae. Table 2 shows the results of the activity of three extracts, and it can be seen that EtOAc was presented as the most promising of them. Although CH_2_Cl_2_ and MeOH also showed a larval paralysis percentage above 50%, they had high effective dose (ED_50_) values when compared to the value observed for EtOAc. We observed that EtOAc was able to paralyze 84% of the larvae at a concentration of 1000 µg/ml, whereas CH_2_Cl_2_ and MeOH did not show relevant activity at this same concentration, as it was necessary to perform the test at 5000 µg/ml for these two extracts.

**Table 2:** Activity of *P. chlamydosporia* extracts on *C. elegans* larvae

Extract	Immobility (%) [concentration of 1000 and 5000 µg/ml]	
	Time: 48 h	ED_50_ (µg/ml)
EtOAC	84.0	325.3
CH_2_Cl_2_	55.5	1400
MeOH	66.9	3365

*Notes*: CH_2_Cl_2_ and MeOH were tested at concentrations of 5000 µg/ml because we did not find an effective dose (ED_50_) in the test with a concentration of 1000 µg/ml. We evaluated the motility of larvae 48 h after contact with the extract

**Fig. 1:**
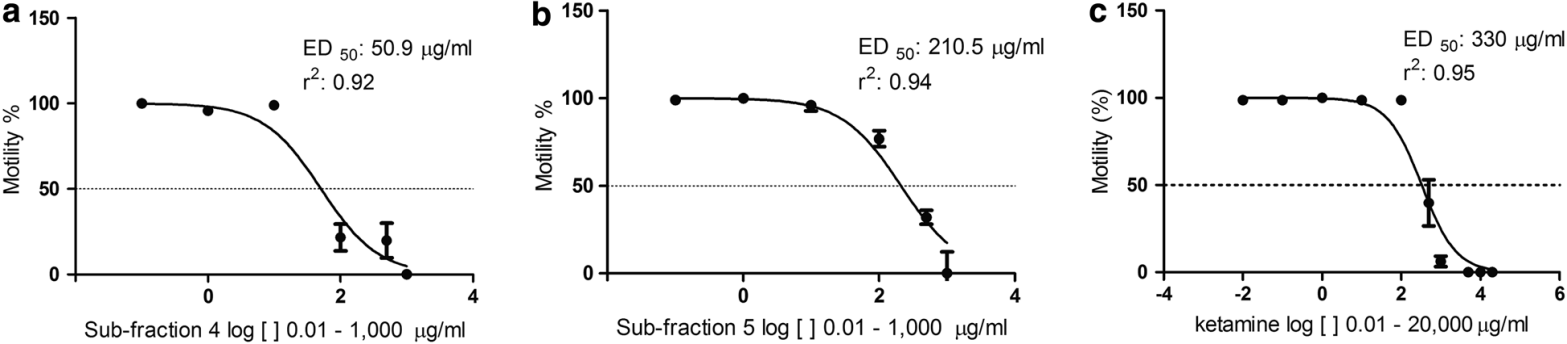
Activity of ethyl acetate sub-fractions 4 (**a**) and 5 (**b**), and ketamine (**c**) on the motility of *C. elegans* larvae. The ketamine was tested until reaching a concentration of 20,000 µg/ml. The motility of larvae was evaluated 48 h after contact with the drug

As EtOAc was the most promising, we fractioned it further, obtaining 7 sub-fractions (Table 1). In the motility test, we observed that only F4 and F5 were active, and it presented the following ED_50_ values: 50.9 μg/ml and 210.5 μg/ml, respectively (Fig. 1). The EtOAc and F4 and F5 reached the following percentages of immobility: 84, 85.5, and 75.7%, respectively, using the concentration of 1000 µg/ml. After the fractionation of EtOAc, the anthelmintic activity remained in F4 and F5.

**Fig. 2:**
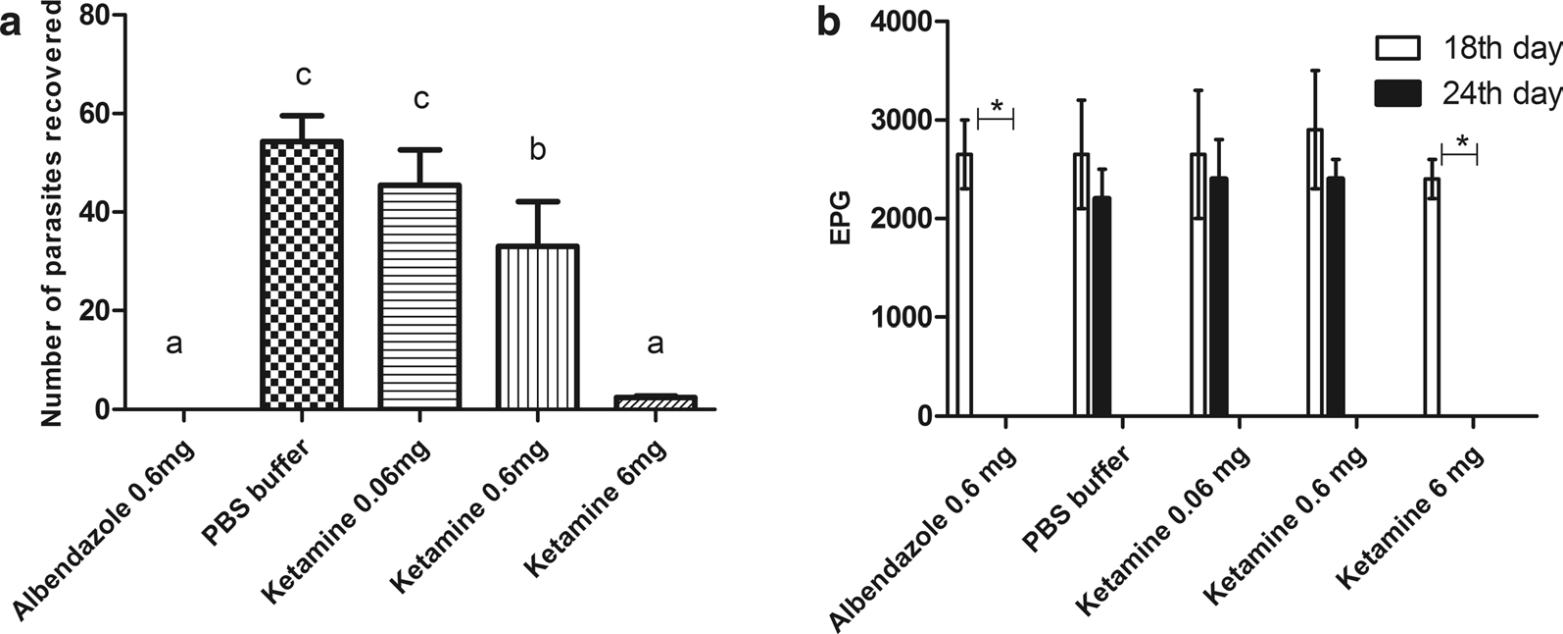
Activity of ketamine on the reduction of *A. ceylanicum* worm burden in hamsters (*Mesocricetus auratus*). **a** Recovered worm burden from hamster intestines. Bars indicated by the same letter signify that there was no statistically significant difference, according to the Tukey’s test (*P* ˃ 0.05). Results were plotted as the mean ± standard deviation. **b** Mean EPG values of the animals in their treatment groups, before (18th day) and after (24th day) the treatment had begun, significant differences, according to Bonferroni correlation (*P* < 0.01), were only present in the albendazole 0.6 mg and ketamine 6 mg

Unfortunately, the amount of biomass produced by the fungus was very small, not being possible to isolate ketamine in sufficient quantity to perform an *in vivo* test using animal models. Therefore, we decided to perform this test using only commercial ketamine on *C. elegans* larvae. Figure 1 shows the activity of the drug on *C. elegans*. As the commercial ketamine was active on the nematode model, we tested it *in vivo* on the animal models infected with *A. ceylanicum*, and *A. suum*. Figure 2 shows that ketamine exhibited activity on *A. ceylanicum* at the concentrations of 0.6 and 6 mg, and the concentration 6mg reduced worm burden parasites to approximately zero; thus, there was no difference (*P* ˃ 0.05) between the groups treated with albendazole (0.6 mg) and ketamine (6 mg). The EPG of the groups treated with albendazole (0.6 mg) and ketamine (6 mg) was zero after 4 days of treatment (Fig. 2).

**Fig. 3:**
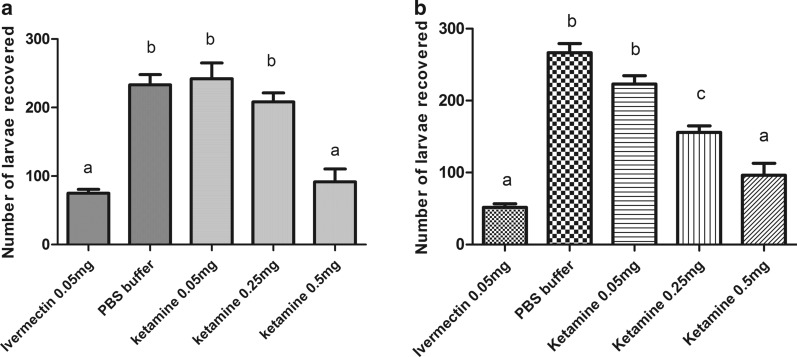
Activity of ketamine on the reduction of the *A suum* larvae burden in lungs of mice (C57Bl6). **a** Administration of drugs by one subcutaneous route. **b** Administration of drugs by oral route. Bars indicated by the same letter signify that there was no statistically significant difference, according to the Tukey’s test (*P* ˃ 0.05). Results are plotted as the mean ± standard deviation

Figure 3 shows the ability of ketamine to impair the infection of *A. suum* larvae in C57Bl/6 mice. The mean number of larvae recovered in the lungs of these animals are shown in this figure. Ketamine showed nematicidal activity in the two routes of the drug’s administration, oral and subcutaneous, with no difference (*P* ˃ 0.05) between the administration routes. In addition, when ketamine was administered orally or subcutaneously, there was no difference in the number of larvae recovered (*P* ˃ 0.05) between ketamine (0.5 mg) and ivermectin.

### Chemical characterization of *P. chlamydosporia* extracts

^1^H NMR analysis of these samples (Additional file 1: Figure S1) revealed characteristic signals of fatty acids and aromatic compounds in EtOAc. Using the GC/MS technique, we could identify the main substances present in EtOAc (Table 3). In sub-fraction 4, we identified ketamine and the acids *cis*-7-hexadecenoic, palmitic, and linoleic acid; however, there were no identified substances other than ketamine in F5 (Additional file 1: Figure S2). The sub-fractions F1, F2, F7, F8, and F9 presented the same fatty acids but not ketamine (Additional file 1: Table S1).

**Table 3:** Substances present in the ethyl acetate fraction identified by GC/MS

Substance	Retention time (min)	Percentage
Ketamine	25.47	15.0
Cis-7-hexadecenoic acid	25.96	0.6
Palmitic acid methyl ester	26.30	26.3
Palmitic acid	27.20	3.6
Linolelaidic acid methyl ester	29.55	26.3
Oleic acid, methyl ester	29.65	23.8
Stearic acid, methyl ester	30.11	3.0
Linoleic acid	30.37	0.9
*cis*-vaccenic acid	30.47	1.9

Ketamine was present in the EtOAc fraction and in sub-fractions F4 and F5 only, and these sub-fractions showed paralyzing activity on *C. elegans* larvae. As some CG/MS analyses revealed greater purity in F5, we chose to perform two-dimensional NMR analyses for this sample. We confirmed the presence of ketamine F5 by HSQC, HMBC, and COSY (Additional file 1: Table S2, Additional file 1: Figures S3–S6 and by UPLC-ESI-MS/MS techniques (Additional file 1: Figures S7, S8), and we compared it to the standard, commercially available ketamine (Table 4 and Additional file 1: Figure S7). We identified ketamine by UPLC-MS analyses, using ESI in the positive mode, through the ions at *m/z* = 238.17 Da [M + H]^+^ and with *m/z* = 240.10 Da [M + ^37^Cl] ^+^. In addition, we observed the spectral ions daughters profile generated from the fragmentation of ions at *m/z* 238.17 Da and 240 Da (Table 4).

**Table 4:** Identification of ketamine in EtOAc fraction by UPLC-ESI-MS/MS

Substance	Retention time (min) UV light^a^	Retention time (min) TIC^+^	[M + H]^+^ *m/z* (Da)	*m/z* of fragments of [M + H]^+^ (Da)
Reference ketamine	2.1	2.3	238.17	179.09, 207.22, 220.13, 238.16
			240.15	127.15, 153.94, 208.98, 222.25, 239.48, 240.05
EtOAc fraction	2.2	2.3	238.17	179.22, 192.28, 207.03, 220.26, 238,03
			240.10	126.83, 154.13, 209.23, 222.06, 239.92, 240.11

^a^Obtained at 220 nm

## Discussion

There are few investments in the discovery of new drugs for the treatment of parasites. Considering that there are reports in the literature relating nemathophagous fungi extracts with nematicidal activity and commercial products using nemathophagous fungi [7, 18, 19], we performed extraction of the *P. chlamydosporia* metabolites with an organic solvent; the extract was fractionated with solvents of different polarity scale and evaluated the compounds for nematicidal capacity. We observed that EtOAc was able to paralyze 84% of the *C. elegans* larvae at a concentration of 1000 μg/ml, whereas CH_2_Cl_2_ and MeOH did not show any relevant activity at the same concentration, which was probably because of the profile and quantity of substances present in EtOAc. The analyses by ^1^H NMR and GC/MS showed the presence of fatty acids and aromatic compounds in EtOAc and CH_2_Cl_2_; however, the GC/MS analysis showed that, in addition to these compounds, ketamine was present in EtOAc. The other substances present in this fraction were the following fatty acids: hexadecenoic; palmitic; linolelaidic; oleic; linoleic; and vaccenic. The palmitic, oleic, and linoleic acids have the previously described nematicidal activity [19, 20]. The other fatty acids are most likely the constituents of the fungal structures, such as the cell membrane. In F4, we identified ketamine and palmitic and linoleic acids, as these fatty acids have nematicidal activity that has already been described in the literature [19, 20], and this can explain the fact that sub-fraction 4 had the lowest ED_50_ compared to sub-fraction 5 and commercial ketamine; thus, the substances could be acting in synergy. On the other hand, F5 indicated only the presence of ketamine. Although the role of fatty acids in nematode fungus interaction is not totally understood, some of these compounds can be present in the formation of adhesive traps in predatory fungi, with the example of linoleic acid [21]. However, the role of such metabolites may go beyond the formation of adhesive traps, since *P. chlamydosporia* does not form such structures.

The GC/MS analysis of F5 suggested the presence of ketamine with a high degree of purity, since it presented a single peak with a retention time of 25.5 min (Additional file 1: Figure S2). The main peak observed by UPLC-ESI-MS/MS analyses displayed a retention time of 2.1 min, with a mass/charge ratio at *m/z* 238.17 Da and 240.10 Da. These ions are the adducts protonated: the ion 238,17 with ^35^Cl isotope and 240.10 with the ^37^Cl isotope. The MS/MS spectra corroborate the presence of ketamine in the samples due to the profile of the ions observed (Table 4, Additional file 1: Figures S7, S8), and its structure was confirmed by 2D NMR analysis (Additional file 1: Figures S3–S6).

Due to the fact that nematicidal activity was observed in EtOAc fraction and F4/F5 sub-fractions, all possessing ketamine, and this molecule were commercially available, we confirm the anthelmintic activity of ketamine on *C. elegans*; thus, we demonstrated that besides the fatty acids with anthelmintic activity known there, ketamine is responsible for the nematode death/paralysis process. Although, the fungi paralysis mechanism on the nematode can occur due to a possible synergy among the substances present. According to Olthof & Estey [22], fungal metabolites capable of paralyzing and/or killing nematodes are involved in the first contact between the fungus and the nematode, so it can be the first method of immobilization, which explains the production of ketamine by the fungus.

The anthelmintic activity of ketamine was confirmed *in vivo* using hamsters and BALB/c mice. Hamsters are permissive hosts for *A. ceylanicum* infection, and the pathogeny caused by these worms in hamsters is similarly caused in humans [23]. The BALB/c mice have been a good alternative model for studying early *Ascaris* spp., and initial events of infection and immunology are similar for the hosts of *A. suum* and *A. lumbricoides* [24]. Ketamine was effective in reducing the burden parasites recovered from the hamster intestines. At a concentration of 6 mg, the worm burden was close to zero, showing results similar to albendazole. In the experiment with *A. suum*, we observed reduction in the number of larvae that reached the mice lungs, and the drug activity was not dependent on the route of administration, since there was no difference in results when the drug was administered subcutaneously or orally. This information is important because the oral route of administration is cheaper, easy to administer, and painless. Possibly, the activity of this drug is related to its ability to bind to GABA receptors and may consequently lead the nematode to paralysis [25, 26]. There is no report in the literature on the anthelmintic activity of ketamine. It is worth noting that this drug is already used as a dissociative anaesthetic. Therefore, for its use as an anthelmintic, more studies are needed to avoid or minimize adverse reactions. Other studies are necessary to verify a better form of administration and/or association with another drug or vehicle once this drug is rapidly metabolized, having a half-life of approximately 15 minutes [27].

## Conclusions

The unprecedented results presented here confirm the anthelmintic activity of ketamine in the tested models, thus demonstrating the possibility of repositioning ketamine and corroborating the potential of *P. chlamydosporia* in the discovery of new drug candidates that can be used in the treatment of several neglected diseases caused by nematode infections.

## Supplementary information

**Additional file 1: Figure S1.** ^1^H NMR spectrum of MeOH, EtOAc and CH_2_Cl_2._ **Figure S2.** GC-MS chromatogram of F5. **Table S1.** Substances present in the sub-fractions F1, F2, F7, F8 and F9. **Table S2.** One and two dimensional NMR spectral data of F5 and its correlations to structure ketamine. **Figure S3.** Key HMBC and COSY correlations of Ketamine. Spectrum of F5 in 2D MNR. **Figure S4.** ^1^H-^1^H COSY, **Figure S5.** ^1^H-^13^C HSQC. **Figure S6.** ^1^H-^13^C HMBC. **Figure S7.** UPLC-ESI-MS/MS of F5. **Figure S8.** Proposal of fragmentation the ion *m/z* 238.17 Da.

## Data Availability

Data supporting the conclusions of this article are included within the article and its additional file.

## Abbreviation

GABA: Gamma-AminoButyric Acid
