# Supplementary material for: Ketamine can be produced by Pochonia chlamydosporia: an old molecule and a new anthelmintic?
Source: Parasit Vectors. 2020 Oct 20;13:527. doi: 10.1186/s13071-020-04402-w (PMC7574484; doi:10.1186/s13071-020-04402-w)
Supplement: Supplementary file 1 — Additional file 1: Figure S1. 1H NMR spectrum of MeOH, EtOAc and CH2Cl2. Figure S2. GC-MS chromatogram of F5. Table S1. Substances present in the sub-fractions F1, F2, F7, F8 and F9. Table S2. One and two dimensional NMR spectral data of F5 and its correlations to structure ketamine. Figure S3. Key HMBC and COSY correlations of Ketamine. Spectrum of F5 in 2D MNR. Figure S4. 1H-1H COSY, Figure S5. 1H-13C HSQC. Figure S6. 1H-13C HMBC. Figure S7. UPLC-ESI-MS/MS of F5. Figure S8. Proposal of fragmentation the ion m/z 238.17 Da. [file 13071_2020_4402_MOESM1_ESM.docx]

**
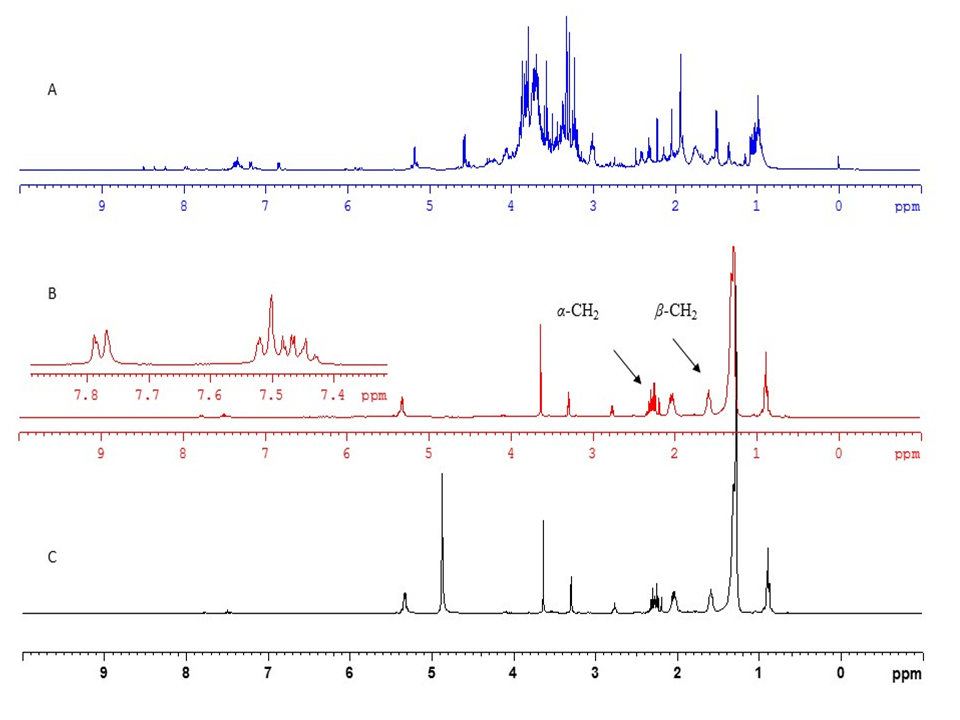
Additional file 1**

**Figure S1** ^1^H NMR spectrum (400 MHz) of (A) MeOH in methanol-*d*_4_ and KH_4_PO_4_ solution in D_2_O; pH 6; (B) EtOAc in CDCl_3_ and (C) CH_2_Cl_2_ in CDCl_3_. The signals at *δ* 1.60 and *δ* 2.30 ppm are characteristics of *β-*CH_2_ and *α*-CH_2_ of saturated fatty acids. Signals of unsaturated fats acids at *δ* 2.07 and *δ* 5.35 ppm. The aromatic compounds appeared between *δ* 7.0 and 8.0 ppm.

**Figure S2** Chromatogram of the F5 which was obtained by gas chromatography coupled to mass spectrometry. The main substance, identified as ketamine, was ionized by electron impact at 70 eV.

| Coding name sub-fraction | Substances |
| --- | --- |
| F1 | Nonanoic acid, 9-oxo-methyl ester; Palmitic acid methyl ester; Oleic acid methyl ester; Stearic acid methyl ester |
| F2 | Nonanoic acid, 9-oxo-methyl ester; Palmitic acid methyl ester; Oleic acid methyl ester; Stearic acid methyl ester; *cis*-Vaccenic acid |
| F7 | Benzeneacetic acid; 2,5-Dihydroxyphenylacetic acid; 1,9-Decadiyne; Benzeneacetic acid, 10-undecenyl ester; Palmitic acid |
| F8 | Linolelaidic acid, methyl ester; Palmitic acid |
| F9 | Palmitic acid; Oleic acid methyl ester; *cis*-vaccenic acid |

**Table S1**  Substances present in the ethyl acetate sub-fractions identified by GC/MS.

**Table S2** ^1^H and ^13^C NMR spectral data and ^1^H-^1^H COSY; ^1^H-^13^C HSQC and ^1^H-^13^C HMBC of F5 and its correlations to structure ketamine.

| Position | *δ* ^1^H^a^ | *δ* ^13^C^b^ | COSY | HMBC |
| --- | --- | --- | --- | --- |
| 1 | - | 208.9 | - | - |
| 2 | - | 70.6 | - | - |
| 3 | 1.83, 2.93 | 38.4 | H-4 | C-2, C-4,C-5, C-1’ |
| 4 | 1.74 | 21.8 | H-3, H-5 | C-5 |
| 5 | 1.86, 2.04 | 28.7 | H-6 | C-4 |
| 6 | 2.52 | 39.8 | H-5 | C-1 |
| 1’ | - | 135.6 | - | - |
| 2’ | - | 134.0 | - | - |
| 3’ | 7.38 | 131.4 | H-4’ | C-5’ |
| 4’ | 7.29 | 129.4 | H-3’, H-5’ | C-2’, C-6’ |
| 5’ | 7.35 | 127.0 | H-6’ | C-1’, C-3’ |
| 6’ | 7.60 | 129.8 | H-5’ | C-4’, C-2’ |
| NCH_3_ | 2.14 | 28.8 | - | C-2 |

a ^1^H determined from HSQC experiment.

b ^13^C determined from HSQC and HMBC experiments.


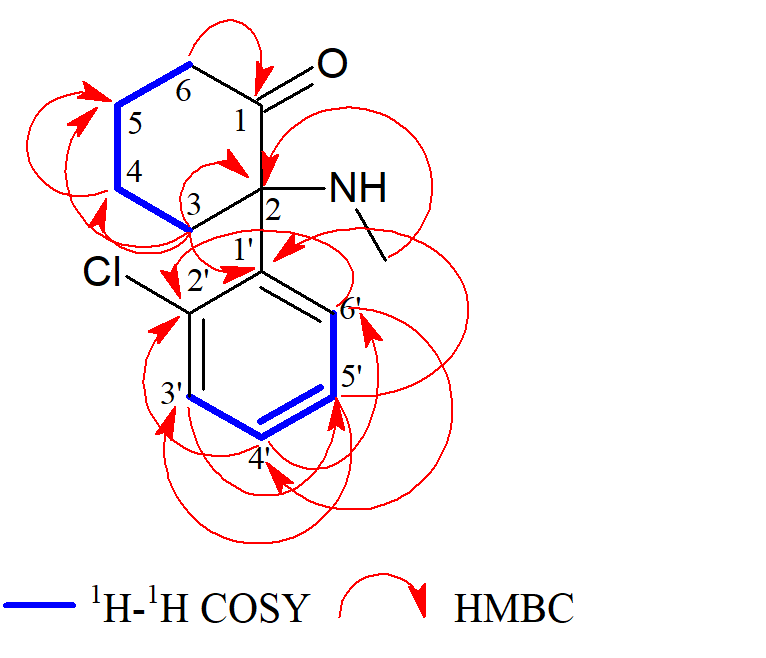


# Figure S3 Key HMBC and ^1^H–^1^H COSY correlations of ketamine.


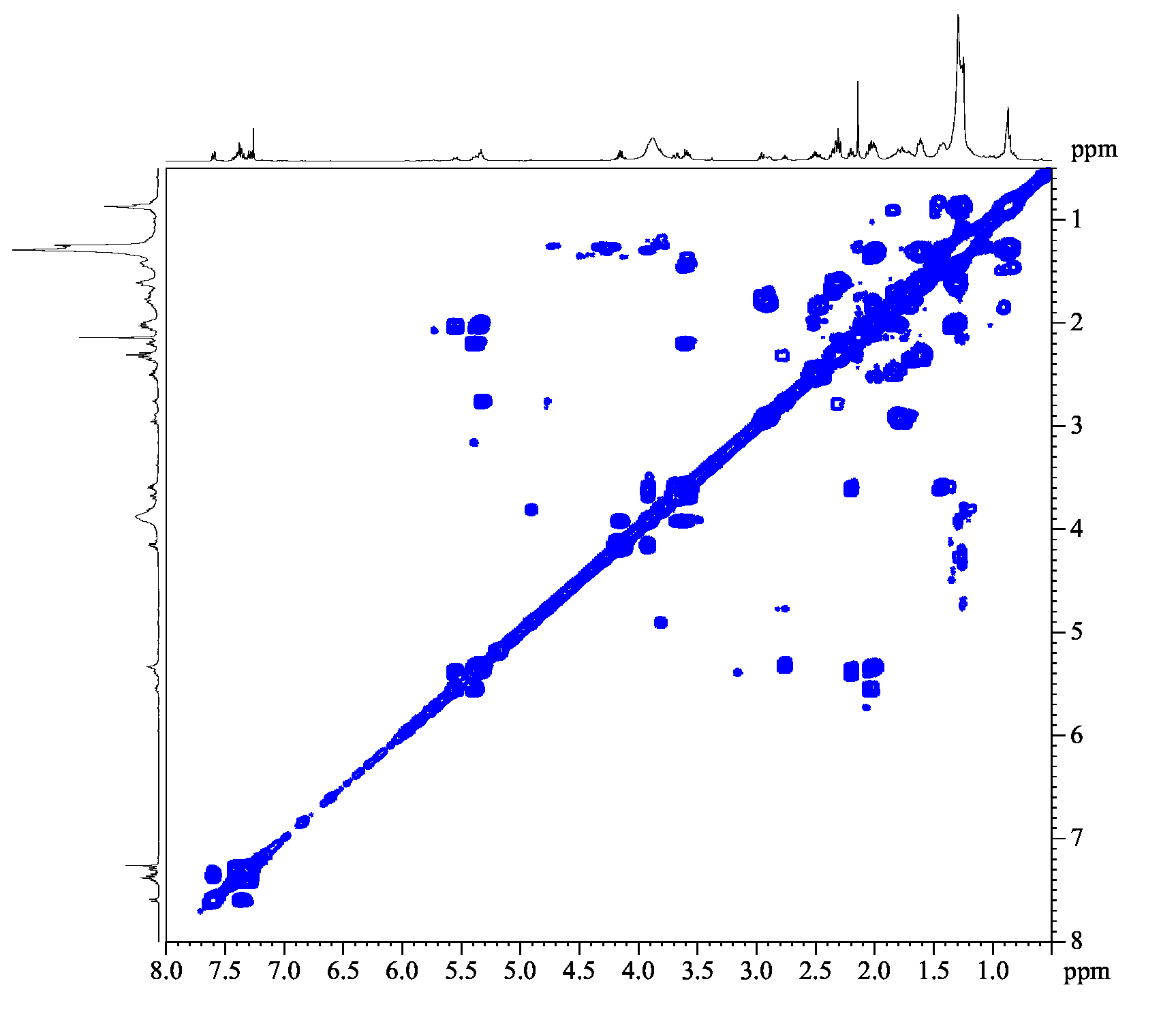


**Figure S4** The ^1^H–^1^H COSY spectrum of F5 in CDCl_3_ at 400 MHz.


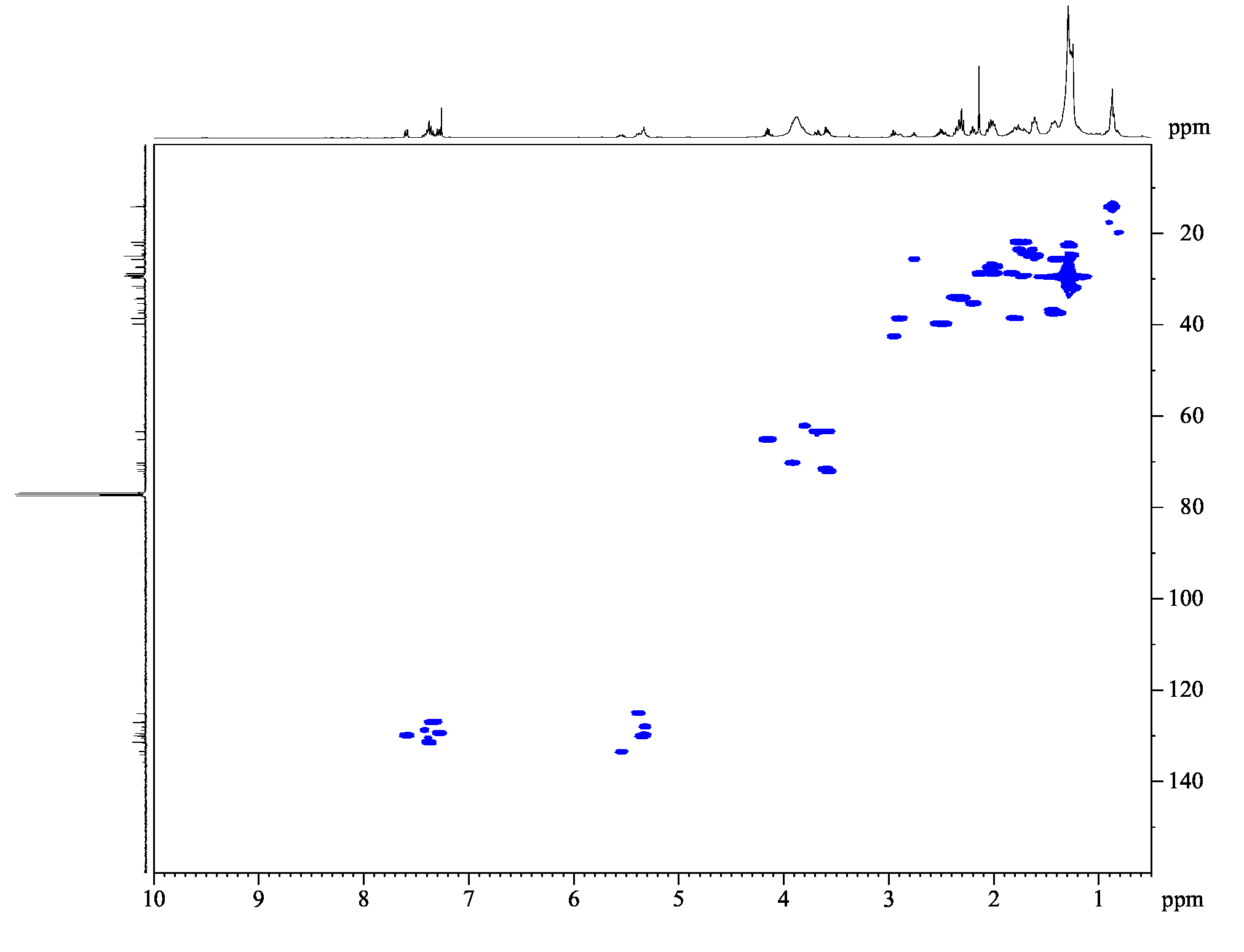


**Figure S54** The ^1^H-^13^C HSQC spectrum of F5 in CDCl_3_ at 400 and 100 MHz.

**
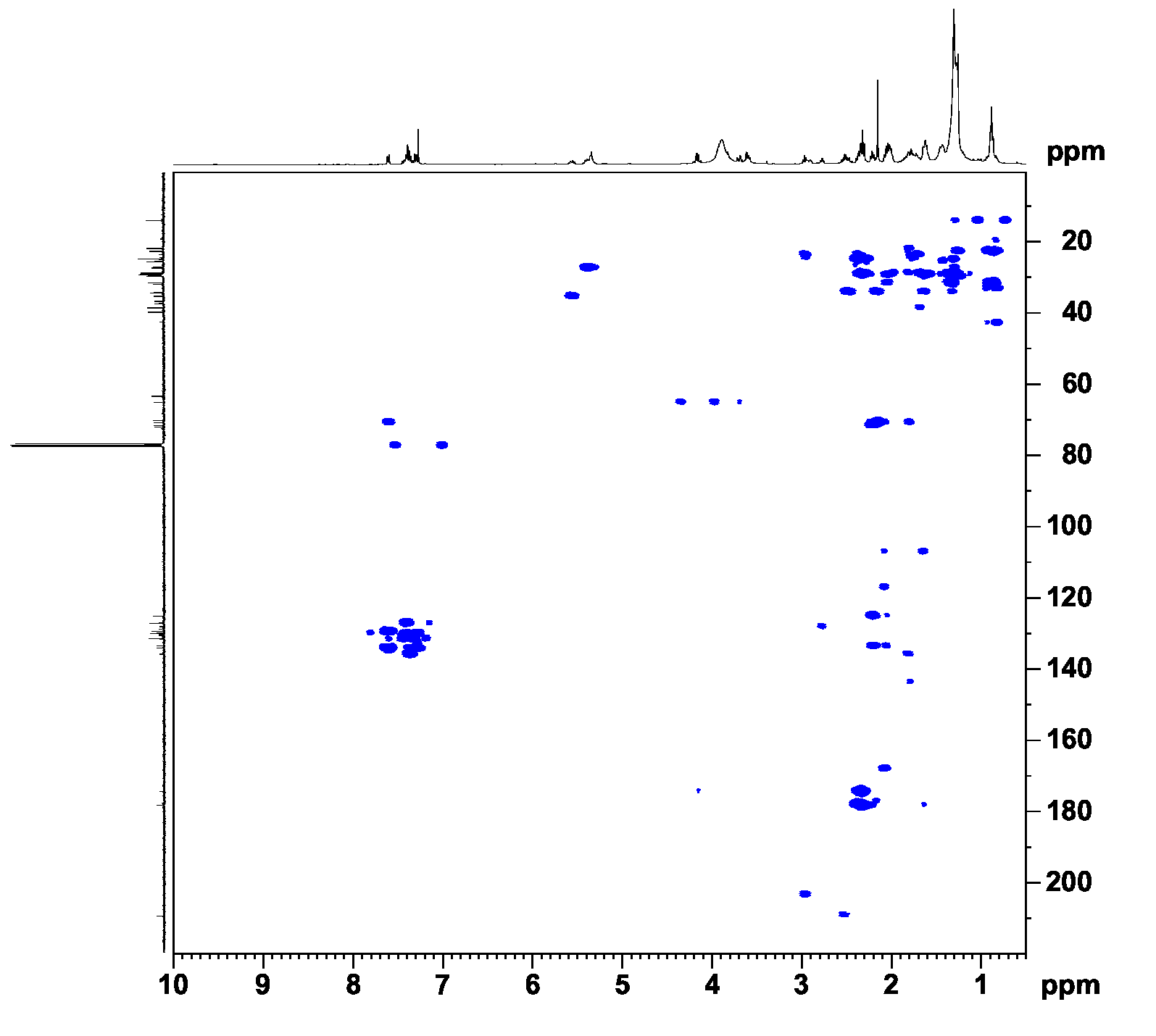
**

**Figure S6** The ^1^H-^13^C HMBC spectrum of F5 in CDCl_3_ at 400 and 100 MHz.


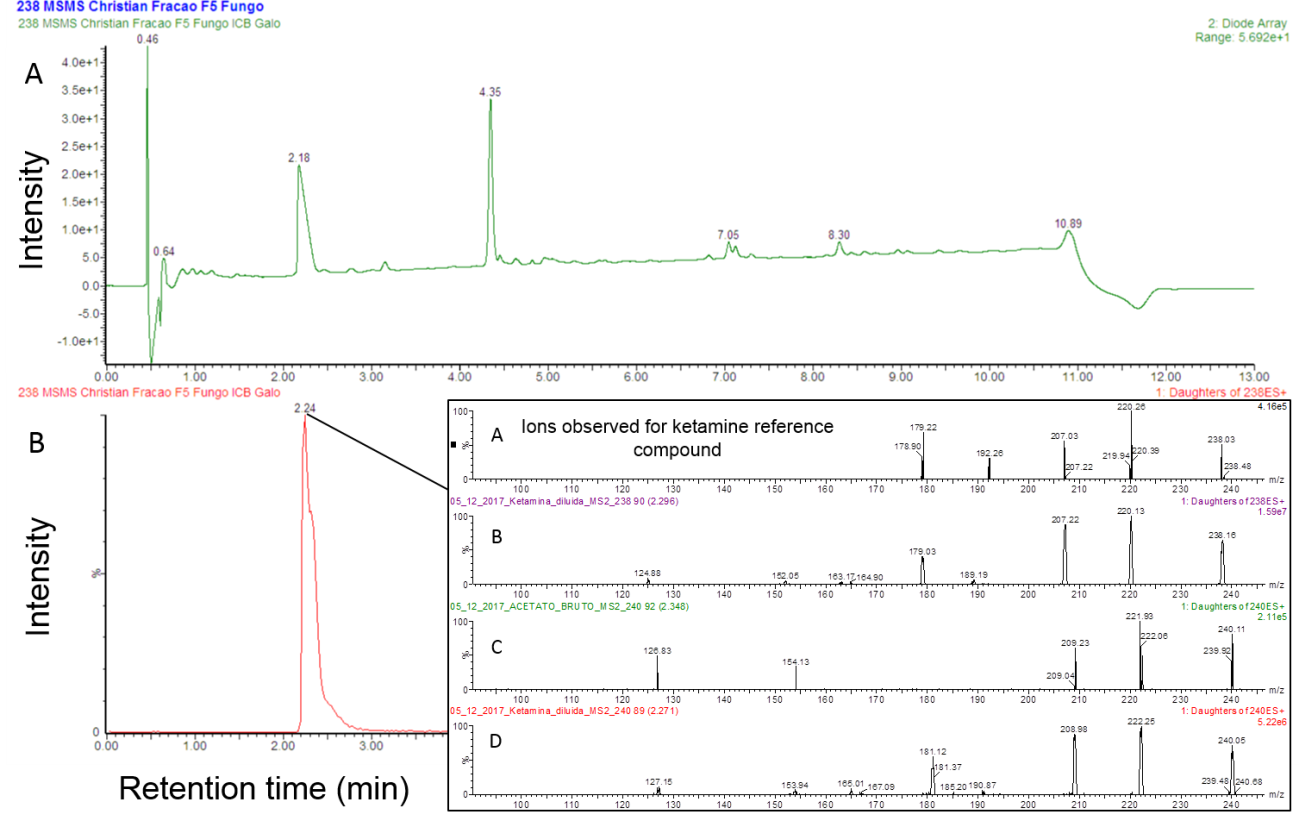


**Figure S7** Chromatogram of EtOAcF5, obtained by UHPLC / MS monitored by ultraviolet light (A) and monitored by positive mode electrospray ionization (B). Spotlight figure spectral profiles fragments obtained in MS/MS mode positive ionization for ions *m/z* = 238 Da (A- EtOAc fraction and B- Ketamine reference) and *m/z* = 240 (C- EtOAc fraction and D- Ketamine reference). m/z = 238 Da [M + H]^+^, m/z = 240.10 Da [M + ^37^Cl]^+^


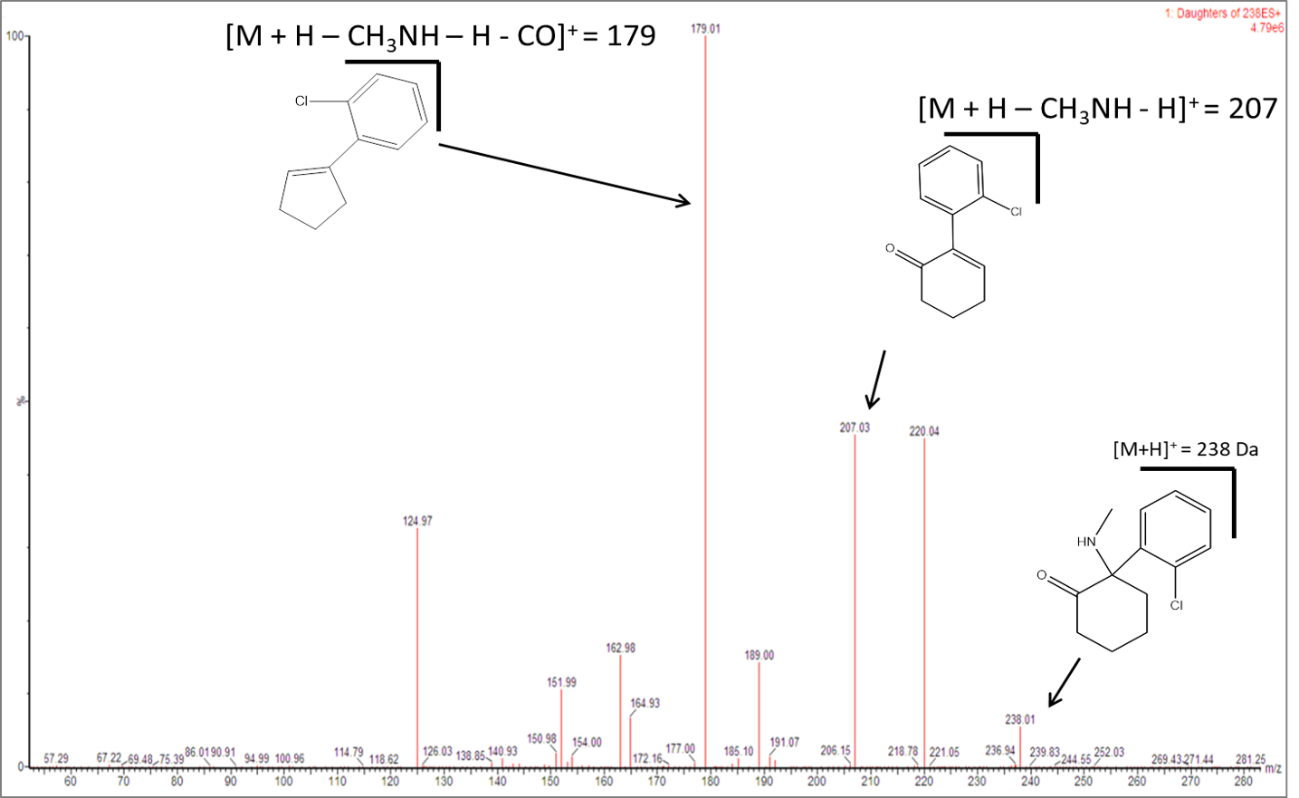


**Figure S8** Proposal of ions after fragmentation the ion *m/z* 238.17 Da, monitored by positive mode electrospray ionization at retention time 2.24 min.
